# Dr. Trotter, on Gastrodynia

**Published:** 1804-10-01

**Authors:** T. Trotter

**Affiliations:** Newcastle


### Medical and Physical Journal.

VOL. XII.] OCTOBER 1, 1804. [NO. LXVIII.

*Printed for R. PHILLIPS, by W. Thorne, Red Lion Court, Fleet Street, London.*

### *To the Editors of the Medical and Physical Journal.*

GENTLEMEN,

THE Case, which I have now the honor to request you will insert in your next Number, I have called GASTRODYNIA; but I shall not dispute the propriety of the term. From the peculiar circumstances under which it was compiled, it claims the attention of the medical world; and I earnestly beg your Correspondents to offer their animadversions upon it, as they regard that liberality of sentiment, and urbanity of manners, which ought to direct the conduct of physicians in their intercourse with one another. What further reflections I may have to offer on this extraordinary incident in my professional exercise, for obvious reasons, must be reserved for a future opportunity. In the mean time, your Readers will give me credit for a small share of passiveness of temper, that I did not provoke a quarrel under the roof of a patient. I have somewhere read, that "a soft answer turneth away wrath, but grievous words stir up anger."

I am, &c.

*Newcastle, Aug.* 1, 1804. T. TROTTER.

---

ON Thursday, Jan. 12, about three in the morning, I was called by the watchman to visit Mrs. ——, in the neighbourhood; a servant girl immediately followed, and called out that her mistress was dying. I found the lady sitting up in bed, and writhing with pain. This happened to be the fifteenth day from her lying-in, of her third child. The pain was felt in the *epigastric region,* towards the *right hypochondrium,* under the last rib, and darting from thence, acutely, upwards to the shoulder of the same side. The pulse about 140, of moderate strength. She had a *diarrhœa* upon her. All circumstances attending the labor had been favorable.

These pains, it appeared, began the preceding evening, had increased gradually till three in the morning, when they became intolerable, and made her cry out with great vehemence. Being an entire stranger to the lady, I enquired of her husband, if, when in her usual health, she was subject to complaints of the stomach, such as, uneasy indigestion, flatulence, constipation of bowels, &c. He answered in the affirmative. I further asked, if gout was known in her family. He said, her father was subject to gout.* And, lastly, I enquired if her medical attendants, at any time, had hinted their suspicion of *gall stones;* or if *jaundice* had ever been noticed. He did not remember any thing being hinted on these subjects.

I gave her immediately Tinct. opii, gt. 30, Mag. ust. ʒß. Sp. menth. pip. ʒij. aa. fontanæ ℥j. ft. haust. In fifteen minutes another draught, with half the quantity of these ingredients, was given. The pain became more endurable, and she ceased to cry out. In half an hour she was considerably relieved; and the pain of the side was only felt on a deep inspiration.

About an hour after my arrival, Dr. Clark and Mr. J. came in; both of whom had seen the lady the night before. I informed these gentlemen of what I had done; and freely imparted to them my opinion of the case. I had just told the husband of the lady, that I conceived the pain to be of the spasmodic kind; depending on debility and an irritable nervous constitution, accompanying the puerperal state. Dr. C. spoke of febrile rigors, cough, and pleuritic stitches, and the danger of puerperal fever; but he gave no decided opinion. It is true, the patient said she had frequent shiverings; was often disagreeably hot, and sometimes in profuse perspirations. But, with such a pulse, how was it possible to suspect a *pleurisy?* These symptoms and feelings only indicated debility, and an accumulated nervous sensibility as peculiar to a lying-in woman; and to be referred to those changes, evolutions, and sympathies in the uterine and mammary organs, after the birth of the infant. A draught with forty drops of tinct. opii was prescribed, lest the pain should return; a blister was

* Two or three days after this, Mr. —— informed me, that his wife's mother was then ill of a complaint which her London physicians called Gout.

was laid on the part affected: A *purging enema* to be followed by an anodyne one, and a *purging mixture,* were also ordered.

About noon the pain returned, and I was sent for. I gave the opiate draught immediately; and ordered a stimulating *enema* of *asafœtida,* &c. to be administered. The pain became easier and died gradually away.

We met in the evening; the patient had three motions in the course of the day, and rather copious. Dr. C. spoke of the propriety of *brisk purgatives.* I saw nothing in the case of the patient that could indicate *brisk purgatives.* The bowels for some days had been very lax, and the lady was much debilitated. Dr. C. would not admit that she was reduced in strength; and said, if you chuse to enquire you will learn that this woman has been indulging in very strong diet. He concluded a long harangue on that subject, by saying, "Your lying-in women constantly eat too much; these London ladies are all full-livers; is your wife a Londoner?"—My wife not being a Londoner, did not come within the pale of his sarcasm. On making enquiry about our patient's food, I was told by the young lady who attended her friend, that it consisted almost of slops, such as tea, coffee, calf's-foot jelly, veal broth, &c.; these in small quantity, and on no day had she been able to eat above half the wing of a fowl. To these articles are to be added three small tumblers of porter; her appetite was certainly much impaired.* All this day the urine was natural.

13th. Friday. About seven in the morning I saw our patient; Mrs. —— had passed a most restless night; her stomach had rejected every thing; and what came up was so sour, that it felt like taking the skin off her throat. Pulse 150, rather low; she complained of great sinking, and said she could not live long in her present condition. I called for a little brandy in a cup; and having inflamed it, I allowed the one half to be consumed. While I was preparing this simple prescription, she discovered great dejection of spirits, and appeared much exhausted. I assured her of immediate relief. She swallowed one mouthful of biscuit soaked in the warm brandy, and with it

U 2 about

* The experienced accoucheur who attended the labour, recommended a nutritive diet, without which he considered so delicate a frame to be unequal to the office of suckling a child.

about four or five tea spoons full of the liquor. These she retained; the vomiting did not return; and in half an hour she took some coffee and toast.

Between nine and ten o'clock the pains of the side and shoulder returned, but not so acutely painful as yesterday. Dr. C. now expressed his opinion that the pain was Pleurisy, and that bleeding was the fittest remedy. Such a declaration astonished me: A pleurisy requiring venæsection, with a pulse beyond 150! I think I heard Dr. Clark say the pulse was 160 by his watch. Blood to about sixteen ounces was taken away: as the first cupful flowed, she said she felt easier, but made no farther expression to that purpose during the bleeding. The pains, as before, declined gradually in the course of the day. I am rather inclined to believe, if Dr. C. had seen this lady at seven in the morning, under the exhaustion, languor, and dejection of spirits, from the sleepless night and constant retching to vomit, that he would have thought bleeding as *little short* of destroying life.

14th and 15th. Saturday and Sunday passed under much uneasiness of bowels, rather than pain, with frequent stools. A draught with colombo and mag. ust. which had been used since Thursday, with an opiate at bed-time, were the chief medicines. Pulse 120; nights restless. The urine, for the last two days, was turbid, as usual where large doses of opium were taken.

I was this day asked by the husband, whether I entertained the same opinion I had at first of his wife's illness. I replied, that I had seen no reason for changing my original sentiments; they were now more confirmed.

16th, Monday. I was called up about four in the morning. I found Dr. Clark and Mr. I. in the house. The pains at the right side and shoulder had returned. Pulse 140. Dr. C. again proposed bleeding; he said it had removed the pain before, and was still the fittest remedy. If I was surprized at the former mention of blood-letting, I was now confounded. There did not appear to me a single symptom to justify it. I asked his explanation. He could give none. I begged he would cite any authority for bleeding in such a case. He answered evasively; but added, that his own practice convinced him he was right. He narrated the case of a Mr. D——ll, where large bleedings had been used; but it had not the slightest analogy to our patient's situation. The common effort of respiration had never been impeded here; there were no dyspnœa, or oppression about the breast; the expectoration was free; nor nor had I ever heard the patient cough during any of my visits. I said, if the disease were pleurisy, as you think, would it not have been aggravated by the large doses of opium taken on the former days? Would you administer opium on the accession, and during the inflammatory stage of pleurisy? He answered, that he had always been in the practice of giving opium from the very first attack, and always observed the *best effects* from it! I reminded him of the lady's naturally weak frame, which I had now learned was of the most delicate cast. I mentioned the debilitating effects of her confinement; the loss of blood which unavoidably attends the most fortunate delivery, as well as what she lost by the former bleeding: I spoke of the very lax state of her bowels for some days past; the acute pain she had suffered; her sleepless nights, and profuse perspirations. Against all these he remained immoveable; and repeated the confidence he gained from his own practice. I went on: Do you then mean to contend, that there is no appeal to first principles in our art? Are the authority of the learned in our profession, the wisdom and experience of ages, to be overthrown by an opinion of either yours or mine, that rests on no foundation? Can you support your method of cure by any practical writer on pleurisy, such as Sydenham, Huxham, or Cleghorn? He offered to appeal to none of these. I quoted the celebrated Aphorism of Hippocrates, in defence of my arguments, "*Qui acidum eructant, non pleuritici sunt.*" If there is a *truism* in medicine, I said, that had obtained the consent of physicians in every age, it is this aphorism; and is exactly in point with our patient's case. He evinced no approbation for the judgement of the Coan Sage; nor did he seem disposed to quit the ground he had taken at the beginning of the conversation. I further asked him, if he thought it were possible for an inflammatory affection of the pleura, requiring blood-letting, to exist in the body with a pulse at 146? He replied, "I allow that the inflammation could not be very active with such a pulse." Then do you call bleeding a passive remedy? "I still think bleeding the best remedy here." This conversation lasted upwards of an hour. The lady admitted that her present pains were by no means so severe as when I first saw her.

About twelve ounces of blood were taken from the arm. The lady was repeatedly asked while the blood flowed, whether the pain of the side felt easier; but she persisted to the last that she felt no relief. Although she lay in the horizontal posture, towards the conclusion she became very faint; but syncope was prevented by volatile salts and a little Madeira wine. The last words the patient uttered as we left the room, were, "*indeed, I feel no easier.*" We retreated indeed without the honor of a triumph. The former blood had been thrown away by mistake; but whether the other gentlemen inspected the last I cannot tell; I heard nothing more about *cupped surface or inflammatory crust.*

Several loose motions took place this day. In the evening I proposed giving wine, and diminishing the opium; it was not agreed to at this visit. The night draught was repeated, and she was ordered the following purgative mixture.

℞. Natron. tart. ℥j. Infus. tamarind. ℥iv. Mannæ ʒiv. M. Sign. A table spoonful of this mixture to be given every two hours till a motion is procured, beginning in the morning, if the bowels have not been opened in the night.

17th, Tuesday. Mrs. —— passed a restless night. She had six motions in the course of this day, resembling coffee grounds, without any mixture of fœcal matter. She complained of great uneasiness of bowels, with much noise and flatulent distension. This day three half glasses of Madeira were taken. Medicines as before.

18th, Wednesday. Very restless last night; bowels uneasy and painful from flatulence. The pains of the side and shoulder returned as before, about eight in the morning. Dr. C. was out of town. To ease the pains, I ordered thirty drops of tinct. opii to be added to the first day draught, and forty drops of æther; and fifteen drops of the tinct. with the same quantity of æther were put in all the other day draughts. A warm plaster was laid over the part affected.

There were languor and depression about our patient this morning which I did not like. I urged her to continue a half glass of Madeira frequently. The pain was soon mitigated, and died away in the course of the day, as it had done before. In the evening she was easy, and appeared disposed to sleep.

At my morning visit, I sent for the cook, and directed her how to prepare beef-tea for her mistress, as strong of the meat as possible. This was done not only for the purposes of nourishment, but as being of an animal nature, was best suited to check that fermentation in the stomach and bowels, that seemed to convert all her other food into flatus flatus and acidity. She took sufficiently of it throughout the day.

19th, Thursday. Mrs. ——— had a good night for the first time, having slept in all six hours. She felt much refreshed. The pulse was now under 100; it was from 96 to 98. I recommended the diet of yesterday to be pursued, and encouraged her with the prospect of a speedy recovery. She had got quit of great quantities of flatus in the night, and felt relief in proportion. The infus. gentian. comp. was ordered in lieu of the colombo.

About noon Dr. C. arrived, not having seen our patient for 40 hours. I informed him, that we had gained much ground in his absence. He asked the usual questions of the lady; and was particularly officious in adjusting the windows for ventilation. He expressed nothing immediately on the favorable change in our patient; but when conversing in the adjoining room, he said to me, "*I firmly believe all cases of this kind would soon recover, were they left to moderate diet, airy rooms, keeping the bowels open, and opiates at bed-time.*" I agreed entirely with him; inasmuch as this treatment excluded all weakening evacuations by *bleeding*, and severe courses of *purging*.

We met in the evening; Dr. C. thought the lady so easy and well, that he would not disturb her with an enema this evening. It is to be observed there was no motion this day! The Doctor seemed in a better humour; he was returning to Durham, to visit a stalled prebend; he shook hands most cordially.

19th, Friday. Our patient had a quiet night. Pulse 104. She had an easy motion this morning; Nature appeared to be resuming her office. Dr. Clark and Mr. I. were both absent at the usual hour of consultation. I directed every thing similar to the preceding day. On Wednesday morning I thought the lady in considerable danger; but now, if the irritable state of the bowels were not to be revived by purgatives, her health and strength must quickly return. I believe the attendants of the lady at this time considered her convalescence certain.

Dr. C. came about noon. He was not satisfied that the patient had only one easy motion; he ordered a saline glyster to be thrown up immediately; three very loose stools followed quickly. I was not present.

We met in the evening. The lady was certainly worse, more irritable and uneasy. Pulse 114. The word *Pleurisy* had not been mentioned since the last bleeding. Dr. C. recurred to his opinion, that purgatives were still the best

 means means of cure; to prevent *Puerperal Fever,* and cleanse the bowels of acrid matter. I said to him, Do you observe any thing that threatens puerperal fever? He gave no direct answer. I repeated, that I saw not the least necessity for purgatives; the uneasy state of bowels here is to be referred to original debility of the intestines; and the irritability of the nervous system is no doubt constitutional, but has been particularly increased by unnecessary bleeding, and too frequent purges. He said in words as nearly as I can recollect, "*I allow that I might have been mistaken, in calling the pain of the side Pleurisy; but long experience in many cases assures me, that purgatives with evening opiates will answer best.*" I agreed with him that the bowels ought to be kept open; but as purgatives must keep the bowels in a constant state of irritation, and drain the body of nourishment, so desirable to be retained under the present circumstances, I could see nothing to justify their exhibition. I proceeded: As you have given up your first opinion, that this lady's disease was *Pleurisy,* what name do you give to her complaint now? "*You mean to oppose my practice; I cannot go on this way; I will leave the patient to you altogether.*" His eyes glistened, and he seemed under considerable agitation when he spoke these words. I was not prepared to answer *unprovoked illiberality;* my attention at the time, was rivetted to the condition of the patient, for whom, in a course of purgatives, I beheld nothing but a train of tortures. Dr. C. went on by saying, that "*Four grains of calomel, to be followed in the morning by a dose of jalap, was the fittest medicine that could be prescribed.*" Such an expression deserved no reply from me, but it made me shudder. I said, that I must maintain my opinion, unless he could produce more satisfactory reasons: if it was to keep the bowels regular, I would agree with him; but purging in so delicate a subject, after a week of suffering, could not be justified. He complained that I differed from him. I answered, that it was as natural for him and I to disagree in opinion, as other physicians had often done before us. Have you not often differed in opinion with Mr. I.? He replied, that they had never materially differed. I mentioned the case of Miss B——, which he had published; and their disputes about the Infirmary; where they had divided the town and neighbourhood on the subject of Fever Wards. The purging medicine that was to begin this *practice,* was as follows:

℞. Sal.

℞. Sal. Ruppell, ʒvj. Lact. amygdal. ℥iv. Sacch. alb. ʒj. M.

Sign. The emulsion. Two table spoons full every hour, till a plentiful easy motion is procured. It is to be recollected, that on this day the patient had a natural easy motion in the morning; three loose evacuations followed the enema; and as we were leaving the house, the nurse announced another. Mr. I. was called up in the night, two more stools having taken place, making in all, *seven* in the space of fourteen hours! I did not see the lady after this evening.

I had now formed the resolution of returning Dr. Clark the compliment, of "*leaving the patient to him altogether*," as he *politely* expressed himself to me. Immediately on coming home, I wrote a note to the lady's husband, requesting he would excuse me from meeting Dr. C. in the morning, and disavowed the treatment about to be adopted. This left Mr. —— to act as he thought proper. It was no longer in my power to do any good; and to receive a *fee* on other terms would have been dishonourable.

This lady, with all the affection of a fond mother, had promised herself the happiness of suckling her child. When I first saw her she had abundance of milk; but on the last evening, the girl who was drawing the breast, complained that it was now so acrid she could scarcely bear it in her mouth. How could it be otherwise under such a draining mode of treatment?

Mrs. —— had formerly suffered severely from piles, as a consequence of weak bowels. Had I been informed of the circumstance, it would have the more confirmed me against all strong purgatives. And indeed, report says, that these tumours did return, and with them swellings of the limbs, requiring the assistance of flannel rollers. I need scarcely add, that by all accounts, this lady's confinement afterwards, was long and painful.

